# Emerging infections: an evolutionary perspective.

**DOI:** 10.3201/eid0403.980306

**Published:** 1998

**Authors:** J Lederberg

**Affiliations:** Rockefeller University, New York, New York, USA.


### Emerging Infections: An Evolutionary Perspective

**Joshua Lederberg**
The Rockefeller University, New York, New York, USA

Our relationship to infectious pathogens is part of an evolutionary drama (1). Here we are; here are the bugs. They are looking for food; we are their meat. How do we compete? They reproduce so quickly, and there are so many of them. They tolerate vast fluctuations of population size as part of their natural history; a fluctuation of 1% in our population size is a major catastrophe. Microbes have enormous potential mechanisms of genetic diversity. We are different from them in every respect. Their numbers, rapid fluctuations, and amenability to genetic change give them tools for adaptation that far outpace what we can generate on any short-term basis.

So why are we still here? With very rare exceptions, our microbial adversaries have a shared interest in our survival. With very few exceptions (none among the viruses, a few among the bacteria, perhaps the clostridial spore-forming toxin producers), almost any pathogen reaches a dead end when its host is dead. Truly severe host-pathogen interactions historically have resulted in elimination of both species. We are the contingent survivors of such encounters because of this shared interest.

### Microbial Resources

### Intraclonal Processes

#### DNA Replication

Microbial intraclonal methods of variation are legion. DNA replication is error prone, and often the constraints of precise replication are turned off in the presence of DNA damage or other injury. Microbes often live in a sea of mutagens, chemical and physical. If we go out in the sun, our skin is damaged; in microbes, UV irradiation goes unimpeded to the very core of their DNA. Those that are not killed are rapidly mutated.

#### RNA Replication

RNA replication is particularly error prone. There are no editing mechanisms for examining the fidelity of replication; therefore, the concept of the quasispecies swarm was recently generated. For many RNA viruses, retroviruses in particular, the rates of mutation are so high that to a close approximation, every particle is genetically different (in at least one nucleotide) from every other particle. They are rapidly evolving as swarms of genotypes, no single genotype being totally representative. Natural selection plays a substantial role. The role of cooperativity in infection of these viruses, particularly among retroviruses and HIV, has not been adequately investigated. Rous sarcoma virus is a case in point. It may be difficult for a single particle, many generations removed from the original competent infector, to consummate an infection by itself, but it can be complemented by other helper viruses present in the same cell.

#### Haploid Organisms

Most of the organisms we are dealing with are haploid, so they have no delay in expressing new genetic factors. The prompt expression may potentially augment cumulative genetic alterations, but in the short run, a resistance mutation will manifest itself almost immediately and will be subject to natural selection very promptly. Multicopy plasmids, which would behave differently, are exceptions.

#### Phase Variation

Phase variation occurs in almost every pathogenic bacterium, in malaria parasites, in trypanosomes, and in Borrelia. Changes that appear to be mutational, on closer examination turn out to be microbial access to an archive of genetic information, much of which has been silenced and then reappears as an adaptive change. The flagellar antigens of salmonella provide the historic example; they can exist in either so-called specific phase or group phase, going back to H1 or H2 loci. We now know that they are the result of silencing one of these loci by the position of a piece of DNA that can be inverted to move the promoter from one locus to another and give a very sudden transformation of the serotype from type 1 to type 2. This is a completely reversible phenomenon; the same event can reinvert that DNA. Many species of site-specific recombinases are capable of scrambling and rescrambling the bacterial genome in order to silence and unsilence genes that may be then carried in an archival state. I pondered why bugs use this mechanism for keeping genes in a cryptic state when gene expression can be (and often is) regulated in other ways. The simplest speculation is that phase variation very often entails controlled antigenic factors. A bug does not want to telegraph to its host in advance that it is carrying even a tiny relic of an alternative epitope because that will provoke immunity on the part of the host even before it has undergone that phase variation.

Genetic factors also control the rates of mutability; whether these factors do or do not directly influence adaptability to virulence is controversial. Preliminary reports suggested that virulent bacteria had a higher incidence of mutators. We now realize that mutators are quite prevalent, and therefore bacteria are constantly facing environmental challenges.

### Interclonal Processes

Recombination mechanisms are quite promiscuous. Conjugation, which can occur between bacteria of widely varying kinds, is most often recognized by plasmid transfer and every now and then by mobilization of chromosomes. Conjugation can even occur across kingdoms, between a bacterium and a yeast, or between a bacterium and a plant. In the case of the rhizobium-like parasite, the crown gall bacterium, genetic material is transferred from the bacterium into the chromosomes of the host plant. Similar phenomena probably occur in eukaryotic infections. Some genes in viruses and bacteria almost certainly were of eukaryotic origin. Some bacteria can deliver DNA intercellularly to their host animals.

Plasmid interchange (movement of tiny bits of DNA from one species to another) is not just a laboratory curiosity; it is the mechanism for rapid spread of antibiotic resistance from widely different species, one to another. It adds even greater cogency to our concerns about the less than optimally advantageous use of antibiotics (e.g., in animal husbandry). The mechanisms exist to make it easy not only for single antibiotic resistance but whole blocks of resistance to be moved from one bacterium to another.

### Host-Parasite Coevolution

Microbes' shared interest in our survival will dominate the overall picture of their evolution. Can this help us predict the outcome of the balance between the host and the pathogen? The possible outcomes are so divergent that it is very difficult to predict in detail what is going to happen in any particular confrontation.

The long-term trend is coadaptation, in which the host acquires factors for resistance and the parasite acquires factors for mitigation and longer survival of (and thereby in) the host. These factors may be genetic mutations, which will certainly be selected.

Other factors include human cultural changes, such as hygienic procedures. The human species outdoes all other species in adopting behavior that is self-destructive rather than self-protective. I am not convinced that every nuance of human behavior has been specifically evolved. Most of our behavior, even the maladaptive self-destructive kind, is learned: the pity and the hope of our species.

Pathogens find it to their advantage to mitigate their virulence, provided they can do so without compromising their livelihood. That is the tightrope they walk. Rhinovirus, the agent of the common cold, is an extremely successful pathogen. We do little to get rid of it. We go to work and school with our runny noses. The virus has a number of adaptations (including the very moderation of its disease process) that tend to facilitate its spread. I worry that a rhinovirus may some day mutate into a somewhat more virulent form, given that it is capable of very rapid spread.

### Evolutionary Strategies

The parasite's dilemma is that if it proliferates rapidly, it may kill the host; that would be a winning strategy if transmission were easy, vectors readily available, the host's behavior obliging, and mosquitoes abundant for high-density spread. Such circumstances are present in northwest Thailand where *Plasmodium falciparum* would be unlikely to survive for very long (because of its profound effects on its host) if the density of spread to new hosts were not favorable. In modern hospitals, the mosquitoes are health-care attendants who inadvertently facilitate the transfer of infection from one patient to another.

### Toxins

It is a wonder that the inexhaustible reservoir of potent toxins has not spread much further. Botulinum toxin, one of the deadliest compounds, is produced in abundance by *Clostridium botulinum*, whose spread to other organisms and potential for becoming a major public health threat can easily be imagined. Why is this toxin so confined? The underlying biologic mechanisms are not confining it; rather, its lethality keeps it under control. The microbe kills its host rapidly, and if it cannot continue to multiply even in the dead host, it reaches a dead end.

In specific physiologic circumstances, these rules of natural selection might not apply. *Escherichia coli* O157 is a case in point. O157 has little to do with *E. coli*; it is a shigella with a little cloak of *E. coli* antigens. O157 should not be used as the sole diagnostic criterion for the spread of shigelloid disease. The toxin genes can inhabit other vectors. The ecologic implications of its human and bovine virulence are not clear. Perhaps polymorphism (changes in bacterial genotype) alters its virulence in human and bovine species. The human loop is quite incidental to its overall survival, as far as we know. The attack rate in humans is only 1%. How has *E. coli* O157 evolved? We understand that as poorly as we understand the sporadic emergence of Legionella from the soil into our air-conditioner ducts.

### Proliferation Rate

If the parasite adopts another strategy and proliferates slowly, we have an evolutionary mechanism in which our own immune system is looking for deviants; this mechanism will be presenting new epitope receptors waiting to be stimulated. Most acute infections produce a full immune response at a humoral and a cellular level within a week or 10 days. So the microbe that proliferates slowly is laying the groundwork for its own vulnerability unless it adopts some further tactics (e.g., phase variation, stealth tactics, antigenic mimicry, exploiting the autotolerance that the host needs to survive its own immune system). Parasites also compete with commensals, with probiotic organisms. This is where HIV runs into severe trouble. Left to its own devices, HIV would not kill its host; but by knocking down the host's immune system, the virus opens the door for other organisms, including commensals, opportunists that can thrive only when the immune defenses are attenuated.

### Symptoms

Vectors are rarely symptomatic, almost never severely symptomatic. The plasmodium would not benefit from killing the mosquitoes that transmit it. If a rabid dog can be considered a vector, its behavioral anomaly illustrates another adaptation that serves the purposes of the parasite.

This line of thinking, what some people have called evolutionary medicine—call it common sense—leads us to look at symptoms. To what extent should we be treating them? Some we treat because they are life-threatening. But is fever, for example, a host defense? Is it a mode of bacterial attack? Is the bacterium or virus producing pyrogens because a higher temperature will promote its own replication? Are pyrogens just side effects of other evolutionary adaptations that have not come to equilibrium? It is hard to avoid models that assume equilibrium; few complex physiologic systems are so obliging. We should question symptoms from an evolutionary perspective. How did they come to be there? This approach may open the door to new avenues of thought in examining the disease process. Cough, diarrhea, or hemorrhage may serve the purposes of the parasite; even so, we may still have to treat hemorrhage, but how far should we go in treating cough? On the one hand, if not too severe, cough may eliminate some of the infectious load from the body; on the other hand, cough generates an aerosol that further disseminates the organism. Cough may have to be treated as a public health measure as much as a therapeutic measure. Diarrhea is another example; it may be a way of eliminating the parasite or a special adaptation enhancing dissemination.

Other symptoms (malaise, headache, pain, itching) probably have different answers. Pain is a puzzling symptom, which plays an indispensable role by drawing attention to a disease. Once the disease is acknowledged, there is no reason in the world not to treat pain. Yet I know of no infection (other than chronic leprosy) that induces anesthesia. It would seem to me that a microbe bent on thriving would impart a sense of euphoria (rather than pain) to its host; we would welcome it and infect ourselves with it. Analgesia may be the eventual moral hazard of biotechnology, the internalized moonshine still or poppy patch.

The ultimate symptom, death of the host, is almost never to the advantage of the parasite. Death signals a breakdown in the equilibrium (the contract between parasite and host) that could have had a better outcome had both sides been more witting.

### Zoonotic Interactions

Many lessons of evolutionary relationships come from zoonotic interactions. Infections that break out of their host of origin often have a very severe impact on their new host. Hantavirus is an outstanding recent example. The pathologic processes in the rodent carriers hardly compare with those in humans. Most zoonotic transfers simply do not work. They are host specific; many are neutral. Every now and then, a zoonotic transfer has enormously larger pathologic implications for the host; these are the transfers we focus on. We presume that the filoviruses and perhaps HIV are in that category. Many, not all, simian immunodeficiency viruses are perceptibly less virulent in their natural host than HIV is in humans, perhaps another example of equilibrium breakdown.

How could the zoonoses be pathogenic when they require so many subtle adaptations to come into a host and really cause disease? Dozens, if not hundreds, of bacterial genes would have to work in concert to be pathogens. Microbes make proteins and carbohydrates, familiar to our systems of immunity. Therefore, if the parasite does not know how to live in the earthly host and the host cannot cope with totally alien parasites, we end up with a wash.

Consider tsutsugamushi fever, scrub typhus. Bangkok is reporting intermediate levels of drug resistance in Orientalia in tsutsugamushi in central and eastern Thailand. The life cycle is one of essentially a hereditary symbiont; the tick is transmitted transovarially and can be communicated from tick to microbe or humans, where it rapidly proliferates. Reinfection back to the tick is not of consequence, which must be a fairly recent spillover of pathogenicity for which there is not ongoing selection. Nothing in the life history of Orientalia would sustain its pathogenicity to maintain its high infectivity.

Years ago planetary quarantine became a policy consideration, beginning with Sputnik in the late 1950s and the early planning of our space program. Would it be permissible to move contaminated spacecraft from one planet to another? Certainly proliferating organisms on Earth could be easily carried to Mars. What would happen if we brought back Mars samples? These considerations resulted in an international convention for the conservation of the microbial virginity of celestial bodies. Sterilization protocols were applied to the Viking Mars spacecraft and by the Russians in the 1970s.

### Maternal Immunity

One mechanism of accommodation is not genetic but physiologic: maternal immunity. The recent outbreak of canine distemper in the lions of the Serengeti (1) demonstrates a quasihereditary cycle that does not involve the genes at all but rather is the propagation of maternal immunity, partial immunity on the part of the offspring, easier adaptation to infection by the host.

### Mitochondria—the Ultimate Pathogens

What are the ultimate pathogens, the ultimate symbionts? The mitochondria. A bacterial invader probably 2.5 billion years ago got into the first eukaryotic cells and conferred oxidative machinery. Who is serving whom? We generally think mitochondria are to our advantage, but think how hard we work to shovel the coal into the furnace that the mitochondria have provided in every cell of our body. Symbiosis is a fact of life, not always friendly or mutually accommodating. In bacteria, plasmids confer great advantages for some functions, but many plasmids also convey a “leave me and you die” message. The plasmid encodes simultaneously for a toxin and an antitoxin but makes sure that the toxin has a longer lifespan. So a bacterium careless enough to drop its plasmid will suffer. The plasmid has the long-term advantage of ensuring that only cells able to continue to proliferate will continue to have the plasmid. So knowing who is serving whom in these kinds of relationships is very complicated.

### Patterns of Evolution

Thanks to the wonders of genomics and DNA analysis, we have a good overall model of the tree of life and the overall patterns of evolution. By the criterion of 16S RNA, extraordinary evolutionary changes have occurred within the multicellular branch, but these changes are not at the level of fundamental housekeeping machinery; they have to do with growing brains, eyes, branches, and flowers, incidental items not at the level of cellular physiology.

### Viruses

Where do viruses come from? Certainly in the world of eukaryotic viruses, no one can say with confidence what the evolutionary provenance is. We believe that viruses originated from some kind of cellular organelle, perhaps ultimately from the nuclear DNA, perhaps from the other organelles. Many of them would have to have undergone enormous changes, and we cannot say which came from where in any tangible example. This complexity can be illustrated (in the prokaryotic systems) by the ease with which viral genomes can be integrated into bacterial chromosomes. These are all double-stranded DNA bacterial viruses, so they have the same fundamental structure as bacterial chromosomes. They go in and out with ease and can be integrated and mobilized, sometimes as viruses, sometimes as bacterial genes. It is impossible to say which came first. If one could point to an evolutionary progression of clusters of genes in a bacterium on the way to generation of a new virus, it would be of some help, but how would one know it was not the relic of a very old one coming back again? Our most fundamental knowledge is very primitive.

### Prions

Prions offer a new paradigm, much of which we do not understand. Stan Prusiner has argued that prions are pure proteins. Trying to understand how a pure protein can propagate confounds our conceptions of the transmission of biological information. So let us say that prion protein (e.g., scrapie prion protein) is a conformational modification of a normal protein, prp-c, coded for by an endogenous gene, a part of the normal genome, not an essential gene. Infected mice show some functional disorders but can survive. One might argue that we do worse with this gene than without it as long as we are susceptible to this modification.

Not much new sequence information is imparted to the normal prion to convert it to the infective agent. The change may be merely in the prion's conformation. We must consider other mechanisms that might cause that same conversion.

The rare nonfamilia incidence of sporadic Creutzfeldt-Jakob disease (CJD) poses a possible example, although it is difficult to exclude some contact with prions in individual cases. We might watch for CJD-like disease as an incident to other kinds of toxic insults. One implication of the protein-prion model, not discussed hitherto, is that conformer alterations may ensure from chemical or physical trauma to preexisting prp-c; heat, toxins, side effects of other infections are candidates (2). Let us carefully label this as wild speculation, pending badly needed assays for this conformer-altering capacity. Other protein-aggregate or amyloid-based diseases (like Alzheimer's) likely have a nucleating episode in their pathogenesis, even if there is no means of contagion from one person to another. At least in the pancreas, amyloid aggregation is a side effect of protein injury by glycation (3).

### Emerging Pathogens

What are we going to do about new, mutant, and recombinant pathogen strains? What can we anticipate about new major outbreaks? How should we be defending ourselves? The good news of course is the wonderful technology in the offing, one marvelous innovation after another in every field of prophylaxis, vaccines, understanding of pathogenic phenomena. The genomics work on bacteria is paying off and may even justify the overall project of human genomics all by itself with its insights into microbial evolution and potential targets for new discoveries in disease management.

At a very high strategic level, we have the basic knowledge to control foodborne epidemics, waterborne epidemics, and fecal-borne diseases. At a technologic level, even sexually transmitted diseases can be controlled. One neglected medium is air. Can we do as well in preventing airborne transmission? Effective control may come down to something as elementary as a face mask like that worn by police in 1918. Control of even a vicious airborne epidemic like influenza should not be above our technical capability. Tens or even hundreds of millions of lives might be at stake over such elementary matters.

The introduction of a new hemolysin into existing anthrax strains in a demonstration of their pathogenicity in golden hamsters (4) required additional epitopes to vaccinate those hamsters against this anthrax. This first example of an artificially contrived new human pathogen illustrates additional challenges in the fight against emerging infections.

Natural infection and disease are enough of a challenge and should not be compounded by human-made agents of death. Biological warfare cannot be endured and must not be tolerated.

Dr. Lederberg, Nobel laureate in physiology or medicine, is a research geneticist, Sackler Foundation scholar, and president emeritus at the Rockefeller University. Dr. Lederberg currently conducts research on genetic exchange mechanisms in bacteria.

### References

1. Lederberg J. Infectious disease as an evolutionary paradigm. Emerg Infect Dis 1997;3:417-23.
2. Causette M, Planche H, Delepine S, Monsan P, Gaunand A., Lindet B. The self catalytic enzyme inactivation induced by solvent stirring: a new example of protein conformational change induction. Protein Eng 1997;10:1235-40.
3. Kapurniotu A, Bernhagen J, Greenfield N, Al-Abed Y, Teichberg S, Frank RW, et al. Contribution of advanced glycosylation to the amyloidogenicity of islet amyloid polypeptide. Eur J Biochem 1998;251:208-16.
4. Pomerantsev AP, Staritsin NA, Mockov YV, Marinin LI. Expression of cereolysine AB genes in *Bacillus anthracis* vaccine strain ensures protection against experimental hemolytic anthrax infection. Vaccine 1997;15(17-18):1846-50.

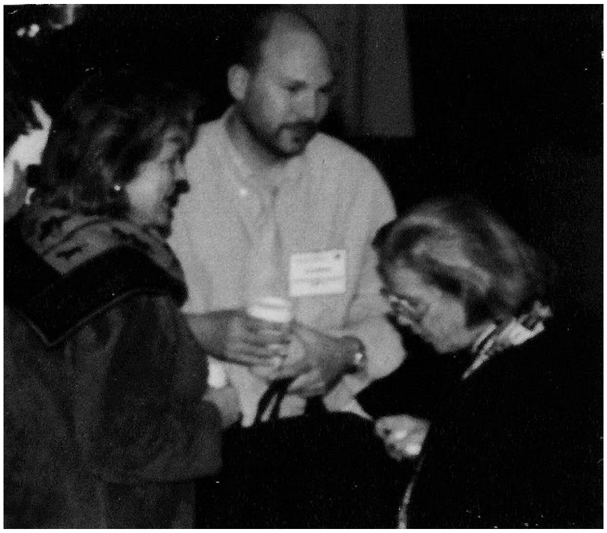

Gail H. Cassell (L)
Lilly Research Laboratories, Indianapolis, Indiana, USA

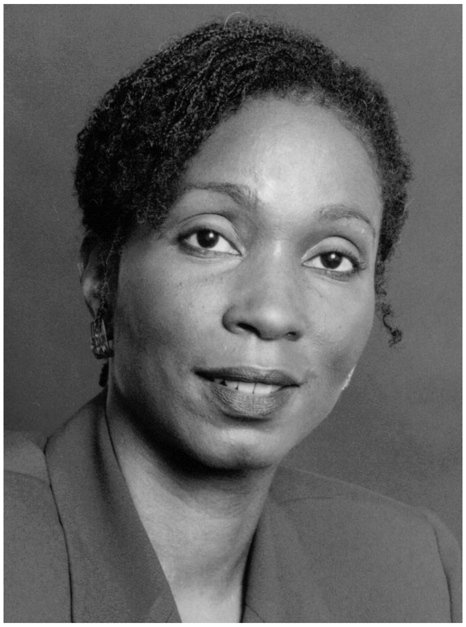

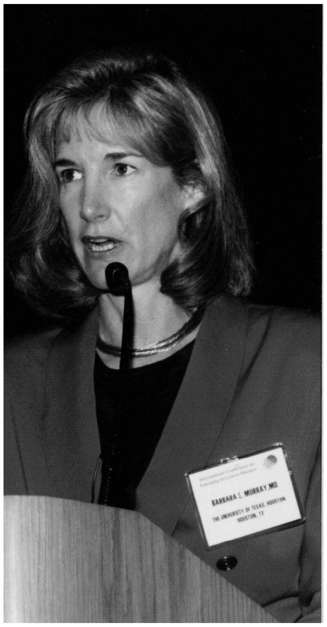

Helene Gayle (L)
Centers for Disease Control and Prevention, Atlanta, Georgia, USA
Barbara Murray
University of Texas Medical School, Houston, Texas, USA
